# Editorial: New advances in obligate intracellular bacteria: pathogenesis and host interactions

**DOI:** 10.3389/fcimb.2023.1338697

**Published:** 2023-11-27

**Authors:** Jun Jiao, Jianfeng Wang, Jiaqi Fu

**Affiliations:** ^1^ Beijing Institute of Microbiology and Epidemiology, State Key Laboratory of Pathogen and Biosecurity, Beijing, China; ^2^ State Key Laboratory for Zoonotic Diseases, Key Laboratory for Zoonosis Research, Ministry of Education, College of Veterinary Medicine, Jilin University, Changchun, China; ^3^ Department of Respiratory Medicine and Center of Pathogen Biology and Infectious Diseases, Key Laboratory of Organ Regeneration and Transplantation of the Ministry of Education, State Key Laboratory of Zoonotic Diseases, The First Hospital of Jilin University, Changchun, China

**Keywords:** obligate intracellular bacteria, pathogenesis, bacteria-host interactions, effector, new advance

Obligate intracellular bacteria (e.g., *Coxiella*, *Ehrlichia*, *Rickettsia*, *Anaplasma*, *Chlamydia*) are an important and fascinating group of microorganisms, as they are often pathogenic to humans and represent a significant public health burden worldwide (Fisher et al., 2021). These bacteria require a host cell for replication and possess mechanisms to secrete effector molecules into the host cytosol, thereby manipulating the processes of host cells (Fu et al., 2022). Recent developments of technology including host cell-free culture system, high-throughput sequencing, and imaging et al. have contributed significant insights into adaptive mechanisms of these bacteria in the intracellular environment. Some of these advances are covered in the present Frontiers Research Topic.

Obligate intracellular bacteria establish infection by manipulating multiple conserved cellular signaling pathways through bacteria-host interactions to subvert innate immune defenses (Walch et al., 2021). Pittner et al. reviewed the extracellular and intracellular roles *Ehrlichi chaffeensis* 120 kDa tandem repeat protein (TRP120) effector SLiM-icry plays during infection - mediated through a variety of SLiMs - that enable *Ehrlichi* to subvert mononuclear phagocyte innate defenses. Since the host long non-coding RNAs (lncRNAs) play a critical role in cancer, immune response regulation, and host-pathogen interaction (Agliano et al., 2019), Arunima et al. discussed the functional roles of lncRNAs in regulation of host immune responses, signaling pathways during host-microbe interaction and infection caused by obligate intracellular pathogens, and summarizes the translational potential of lncRNA research in development of diagnostic and prognostic tools for human diseases. In addition, the host p62 is also crucial in eradicating intracellular bacteria by xenophagy (Lee et al., 2022), Zhou et al. discussed the various roles of p62 in intracellular bacterial infections and how the innate immune system functions in response to obligate intracellular bacteria.

*Mycobacterium* is an intracellular, facultative bacterium known to colonize and infect the human host through ingestion or respiratory inhalation (Brown-Elliott and Philley, 2017). By combining proteomics and metabolic pathways, Abukhalid et al. found a large number of small-molecule metabolites and co-factors which are essential for biofilm formation of *Mycobacterium avium*, and those differential metabolites and the associated metabolic pathways can be regarded as novel targets for the development of biofilm-based treatments and antibiotic discovery against *M. avium* infection. Another article provided by Cheng et al. found that MP3RT, a novel peptide-based vaccine against tuberculosis infection, was a non-toxic and sensitizing vaccine with high antigenicity and immunogenicity *in silico* analysis combining with animal experiments, indicating that immunoinformatic techniques can be generalized in the field of reverse vaccinology.

*Porphyromonas gingivalis* is a gram-negative bacterium and widely regarded as the key pathogen of chronic periodontitis and its outer membrane vesicles (OMVs) are the key in the virulence and pathogenicity (Honda, 2011). A study performed by Mao et al. showed that the protein composition of OMVs isolated from different growth stages demonstrated obvious differences ranging from 25 KDa to 75 KDa, and the late-log and stationary OMVs can boost the formation of M1 macrophages and promoting the expression of inflammatory cytokines in macrophages and stimulate the NLRP3/IL-1β-related pathway of periodontal ligament stem cells (PDLSCs).

*Wolbachia* are common gram-negative intracellular bacteria that are maternally inherited endosymbionts and *Wolbachia* infection is estimated to naturally occur in 66% of known insect species (Beckmann et al., 2017). The most common techonology used for *Wolbachia* detection in a host species is polymerase chain reaction (PCR). By PCR, Zhang et al. firstly reported *Wolbachia* infections in *Aedes aegypti* in China. Besides PCR, metagenomic next-generation sequencing (mNGS) is a high-throughput sequencing technology and can be used to directly sequence nucleic acids in clinical host samples. Fang et al. reported a case of *Chlamydia psittaci* pneumonia combined with Guillain-Barré syndrome detected by mNGS, proving the value for the diagnosis of complex, mixed obligate intracellular pathogens using mNGS.

In conclusion, this Research Topic provided updated knowledge into understanding on the pathogenesis and host interactions of obligate intracellular bacteria. We thank all authors who contributed their works and all reviewers for their time and insightful comments that led to this exciting Research Topic.

## Author contributions

JJ: Writing – review & editing, Writing – original draft. JW: Writing – review & editing. JF: Writing – review & editing.

## References

[B1] Agliano F. Rathinam V. A. Medvedev A. E. Vanaja S. K. Vella A. T. (2019). Long noncoding RNAs in host-pathogen interactions. Trends Immunol. 40, 492–510. doi: 10.1016/j.it.2019.04.001 31053495 PMC6556373

[B2] Beckmann J. F. Ronau J. A. Hochstrasser M. (2017). A *Wolbachia* deubiquitylating enzyme induces cytoplasmic incompatibility. Nat. Microbiol. 2, 17007. doi: 10.1038/nmicrobiol.2017.7 28248294 PMC5336136

[B3] Brown-Elliott B. A. Philley J. V. (2017). Rapidly growing mycobacteria. Microbiol. Spectr. 5 (1). doi: 10.1128/9781555819866.ch41 PMC11687460 28084211

[B4] Fisher J. R. Chroust Z. D. Onyoni F. Soong L. (2021). Pattern recognition receptors in innate immunity to obligate intracellular bacteria. Zoonoses (Burlingt) 1 (1), 10. doi: 10.15212/ZOONOSES-2021-0011 35282331 PMC8909792

[B5] Fu M. Zhang J. Zhao M. Zhang S. Dai L. Ouyang X.. (2022). *Coxiella burnetii* plasmid effector B promotes LC3-II accumulation and contributes to bacterial virulence in a SCID mouse model. Infect. Immun. 90, e0001622. doi: 10.1128/iai.00016-22 35587202 PMC9202392

[B6] Honda K. (2011). Porphyromonas gingivalis sinks teeth into the oral microbiota and periodontal disease. Cell Host Microbe 10, 423–425. doi: 10.1016/j.chom.2011.10.008 22100158

[B7] Lee Y. J. Kim J. K. Jung C. H. Kim Y. J. Jung E. J. Lee S. H.. (2022). Chemical modulation of SQSTM1/p62-mediated xenophagy that targets a broad range of pathogenic bacteria. Autophagy 18, 2926–2945. doi: 10.1080/15548627.2022.2054240 35316156 PMC9673928

[B8] Walch P. Selkrig J. Knodler L. A. Rettel M. Stein F. Fernandez K.. (2021). Global mapping of *Salmonella enterica*-host protein-protein interactions during infection. Cell Host Microbe 29, 1316–1332.e12. doi: 10.1016/j.chom.2021.06.004 34237247 PMC8561747

